# Multivariate analysis of clinical characteristics and prognostic factors in early-onset alopecia areata: a retrospective study with 82 Brazilian patients^☆^

**DOI:** 10.1016/j.abd.2022.11.001

**Published:** 2023-05-08

**Authors:** Isabella Doche, Paula Gerlero, Tiara Magalhães, Chan I. Thien, Thalita Macedo, Maria Cecília Rivitti-Machado

**Affiliations:** Department of Dermatology, Faculty of Medicine, Universidade de São Paulo, São Paulo, SP, Brazil

Dear Editor,

Alopecia Areata (AA) is an autoimmune form of nonscarring hair loss affecting both adults and children. While approximately 2% of the general population develops the disease at some point in their lives, pediatric AA constitutes approximately 20% of all cases, and around 60% will develop the first patch before the age of 20 years.^1^ However, up to 50% of young patients with limited disease usually show regrowth within one year without treatment. Although there are some AA known features considered as poor prognosis indicators,^2^ the course of the disease is still unpredictable and the response to treatment can be variable. Recently, some authors tend to recognize AA as a systemic condition due to the increased association with some comorbidities, especially metabolic and cardiovascular diseases.^3^

The authors aimed to identify and analyze potential clinical characteristics and prognostic factors related to treatment outcomes in early-onset AA. This retrospective study included 82 patients of both genders and all skin types, clinically diagnosed with the first episode of AA before the age of 18 years at the Hair Disease Clinic from Hospital das Clínicas of the University of São Paulo, Brazil, between 2014 and 2019. Patients’ data were retrieved from medical charts and assessed by issuing multivariate analysis. Exclusion criteria included other types of scalp alopecia.

This study included 43 females and 39 males with a current mean age of 20.44 (4‒43) years and a mean age of disease onset of 7.7 years. The mean duration of disease was 10.94 (0.5‒36) years and the mean follow-up was 24.7 (0.5‒60) months. The most frequent associated comorbidity was hypothyroidism (n = 20; 24.4%); however, only 5 patients (6.1%) presented with Hashimoto’s disease overall. The most frequent type of AA was *universalis* (n = 28; 34.1%). Almost 60% (n = 49) of the patients had eyelashes, eyebrows, and/or nail involvement. Almost all patients (n = 79, 96.3%) underwent topical therapy associated or not with other treatments that included intralesional steroids, systemic steroids, cyclosporine, UVB-NB phototherapy and dyphencyprone. Sixty-two percent (n = 51) showed a complete or partial hair regrowth and 23.3% (n = 19) had recurrences after five years, mostly presented as multiple patches, shown in Table 1.

**Table 1:** Demographics, clinical and therapeutic features of the patients

	Median	SD	(Range)	Median	Nº Patients, % (n = 82)
**Gender**					
Male					39 (47.6)
Female					43 (52.4)
**Age (y)**					
Total	20.4	9.2	(4‒43)	17	
Male	17.6	6.2	(6‒33)	17	
Female	23	10.8	(4‒43)	22	
**Age at first visit (y)**					
Total	7.7	4.4	(0‒18)	6.5	
Male	7.6	4.2	(0‒18)	6	
Female	7.7	4.6	(0‒18)	7	
**Duration of disease (y)**					
Total	10.9	8	(0.5‒36)	9.5	
Male	8	5.2	(1‒23)	6	
Female	13.5	9.2	(0.5‒36)	10	
**Follow-up time (mo)**	24.7	16.9	(0.5‒60)	24.5	
**Relapse time (mo)**	87.4	225.1	(6‒1220)	24.4	
**Type of hair loss**					
*Universalis*					28 (34.1)
Multiple patches					24 (29.3)
Single patch					15 (18.3)
*Totalis*					12 (14.6)
*Ophiasis*					3 (3.7)
**Eyelashes, eyebrows and/or nails involvement**					49 (59.8)
**Family history of AA**					6 (7.3)
**Hypothyroidism**					20 (24.4)
**Hashimoto disease**					5 (6.1)
**Atopic dermatitis**					4 (4.9)
**Asthma**					6 (7.3)
**Vitiligo**					4 (4.9)
**Obesity**					4 (4.9)
**Down’s syndrome**					4 (4.9)
**Topical treatment**					79 (96.3)
Corticosteroids alone					63 (76.8)
Minoxidil					22 (26.8)
Tacrolimus					18 (22)
Anthralin					39 (47.6)
**Intralesional steroid**					26 (31.7)
**Diphencyprone**					15 (18.3)
**Systemic treatment**					29 (35.4)
Oral corticosteroids					22 (26.8)
Cyclosporine					68 (82.9)
**Phototherapy (UVB-NB)**					68 (82.9)
**Treatment response (partial or complete hair regrowth)**					51 (62.2)
**Recurrence**					19 (23.2)
**Type of hair loss recurrence**					
Multiple patches					14 (17.1)
*Universalis*					3 (3.7)
*Totalis*					2 (2.4)

AA, Alopecia Areata; SD, Standard Deviation; mo, months; N, Number of patients; UVB-NB, Ultraviolet B-Narrow Band; y, years.

The use of intralesional steroid injections was the only statistically significant variable related to better treatment response in the multivariate analysis, p = 0.007 (OR = 4.56, 95% CI 1.52‒13.6). Intralesional steroid injections using, Triamcinolone Hexacetonide (TH) at the mean concentration of 5 mg/mL (2.5‒10 mg/mL) were done in 26 patients over 13 years old in a monthly basis and the mean number of sessions was 8.7. Of note, patients who underwent TH injections mostly presented with AA *universalis*, had higher mean age, higher age of AA-onset, longer illness, and longer follow-up, compared to the general group. No relevant side effects were reported in this group, except for local pain, minimal puncture bleeding, and transitory skin scalp atrophy.

Current evidence suggests that thyroid dysfunction and autoimmune thyroid diseases are more prevalent in patients with AA,^4^, ^5^ but data in children is still lacking. Although almost half of the patients with hypothyroidism presented with either AA *universalis* or *totalis,* it was not associated with an inferior therapeutic response. Also, known features usually related to a poor prognosis such as having extensive AA (*totalis*/*universalis*), nail involvement, family history of AA, or atopic dermatitis were not related to a worse therapeutic outcome in this study. On the other hand, adherence to treatment was the only modified prognostic factor, as previously reported.^6^

Although there are many treatments for AA, currently available options are not curative, at most control and limit the disease, and relapses are frequent over time. Choosing the proper treatment relies on considering the age of the patient, the extension, and the duration of the disease. According to the AA Consensus of Experts Study, for patients over 13 years old with AA involving 30%‒50% of the scalp, intralesional corticosteroids (preferably Triamcinolone Acetonide ‒ TA) are considered first-line therapy.^7^ TH, marketed as 20 mg/mL sterile suspension, is the most available therapeutic option for intralesional steroid injection in our country. Although TH is thought to have a better efficacy compared to TA,^8^ it is a less soluble derivative of TA and lower doses should be used in order to avoid skin atrophy.^9^

Limitations of this study include retrospective analysis, lack of a control group, and treatment modalities chosen according to availability in our Institution.

Our findings support that consecutive intralesional steroid injections can be a modifying factor in the course of AA helping to promote hair regrowth and treatment adherence, especially in early-onset cases. Also, intralesional injections with TH can be considered an effective, safe, and low-cost therapy for adolescents with AA, if TA is not available.

## Statement of ethics

This study protocol was reviewed and approved by our University Ethics Committee, University of Sao Paulo Medical School, and approval number: 4.919.529.

All the patients in this manuscript have given written informed consent for publishing their case details. For all underaged participants, written informed consent was obtained from all participants' parents/legal guardians/next of kin to participate in the study.

## Financial support

None declared.

## Authors’ contributions

Isabella Doche: Contributed to the study concept and design, the acquisition, analysis, and interpretation of data; contributed to writing the manuscript and giving a critical review of important intellectual content; participated in research guidance, intellectual participation in the therapeutic management of the cases studied and in the critical review of the literature; approval of the final version of the manuscript.

Paula Gerlero: Contributed to the study concept and design, the acquisition, analysis, and interpretation of data; contributed to writing the manuscript and giving a critical review of important intellectual content; participation in research guidance, intellectual participation in the therapeutic management of the cases studied and in the critical review of the literature; approval of the final version of the manuscript.

Tiara Magalhães: Contributed to the study concept and design, the acquisition, analysis, and interpretation of data; participation in research guidance, intellectual participation in the therapeutic management of the cases studied and in the critical review of the literature; approval of the final version of the manuscript.

Chan Thien: Contributed to the study concept and design, the acquisition, analysis, and interpretation of data; participation in research guidance, intellectual participation in the therapeutic management of the cases studied and in the critical review of the literature; approval of the final version of the manuscript.

Thalita Macedo: Contributed to the study concept and design, the acquisition, analysis, and interpretation of data; participation in research guidance, intellectual participation in the therapeutic management of the cases studied and in the critical review of the literature; approval of the final version of the manuscript.

Maria Cecília Rivitti-Machado: Contributed to writing the manuscript and giving a critical review of important intellectual content; participation in research guidance, intellectual participation in the therapeutic management of the cases studied and in the critical review of the literature; approval of the final version of the manuscript; contributed to the study concept and design, the acquisition, analysis, and interpretation of data.

## Conflicts of interest

None declared.
